# Effects of Alpha Particle and Proton Beam Irradiation as Putative Cross-Talk between A549 Cancer Cells and the Endothelial Cells in a Co-Culture System

**DOI:** 10.3390/cancers7010481

**Published:** 2015-03-18

**Authors:** Hélène Riquier, Denis Abel, Anne-Catherine Wera, Anne-Catherine Heuskin, Géraldine Genard, Stéphane Lucas, Carine Michiels

**Affiliations:** 1 URBC-NARILIS, University of Namur, 61 rue de Bruxelles, Namur 5000, Belgium; E-Mails: helene.riquier@gmail.com (H.R.); abel_denis@hotmail.com (D.A.); geraldine.genard@unamur.be (G.G.); 2 LARN-PMR, NARILIS, University of Namur, Namur 5000, Belgium; E-Mails: a.wera@surrey.ac.uk (A.-C.W.); anne-catherine.heuskin@unamur.be (A.-C.H.); stephane.lucas@unamur.be (S.L.)

**Keywords:** radiobiology, hadrontherapy, radioresistance, crosstalk, co-culture, endothelial cells

## Abstract

*Background*: High-LET ion irradiation is being more and more often used to control tumors in patients. Given that tumors are now considered as complex organs composed of multiple cell types that can influence radiosensitivity, we investigated the effects of proton and alpha particle irradiation on the possible radioprotective cross-talk between cancer and endothelial cells. *Materials and Methods*: We designed new irradiation chambers that allow co-culture study of cells irradiated with a particle beam. A549 lung carcinoma cells and endothelial cells (EC) were exposed to 1.5 Gy of proton beam or 1 and 2 Gy of alpha particles. Cell responses were studied by clonogenic assays and cell cycle was analyzed by flow cytometry. Gene expression studies were performed using Taqman low density array and by RT-qPCR. *Results*: A549 cells and EC displayed similar survival fraction and they had similar cell cycle distribution when irradiated alone or in co-culture. Both types of irradiation induced the overexpression of genes involved in cell growth, inflammation and angiogenesis. *Conclusions*: We set up new irradiation chamber in which two cell types were irradiated together with a particle beam. We could not show that tumor cells and endothelial cells were able to protect each other from particle irradiation. Gene expression changes were observed after particle irradiation that could suggest a possible radioprotective inter-cellular communication between the two cell types but further investigations are needed to confirm these results.

## 1. Introduction

Radiotherapy plays an important role in cancer treatment since more than 50% of all cancer patients will receive at least one session of radiation therapy during their treatment [1]. Unfortunately, the total dose given to the tumor is usually limited by the tolerance of healthy tissues surrounding the tumor. To improve radiotherapy, the dose given to the tumor must be maximized while minimizing the radiation effect on normal tissues. In this way, new radiation techniques, such as intensity-modulated radiation therapy (IMRT) and hadrontherapy, were developed to improve the dose delivery to the patient [2]. Hadrontherapy is an emerging technique in radiation therapy that uses positively charged particles instead of conventional photons. The use of charged particles in radiotherapy has several advantages over X-rays, the most important of which is the more precise dose distribution within the target volume. For this reason, charged particles are already used to treat paediatric patients and tumors located near critical healthy tissues, such as melanoma of the eye, chordomas and chondrosarcomas at the base of the skull [3]. Because of their high linear energy transfer (LET), heavy charged particles, such as carbon or helium ions, produce an intense ionization along their track and cause severe DNA damage which is more difficult to repair than that caused by sparsely ionizing radiation like X-rays [4]. This confers to high-LET charged particles other advantages over X-rays: they have a higher relative biological effectiveness (RBE) and this effectiveness is less dependent on cell cycle position and on tumor oxygenation. As a result, charged particles are more suitable to treat hypoxic and slowly dividing radioresistant tumors [5].

However, the improvement of radiotherapy effectiveness also requires a better understanding of radioresistance mechanisms. Indeed, tumors are now considered as complex tissues composed of different cell types that can influence tumor response to cancer treatment, including radiotherapy [6]. As tumor vasculature is needed to sustain tumor growth, the destruction of the tumor vascular network by ionizing radiation can lead to an enhanced tumor cell killing [7]. However, tumor cells can protect endothelial cells, which constitute tumor blood vessels, from ionizing radiation by secreting growth factors and cytokines that decrease endothelial cell radiosensibility, eventually leading to tumor radioresistance due to the limited effect of ionizing radiation on tumor vasculature [8,9,10]. Conversely, it has been demonstrated that tumor-associated endothelial cells can enhance tumor cell proliferation and protect them from ionizing radiation [11,12,13,14]. This radioresistance-inducing dialog has been demonstrated using X-rays but very little is known regarding this issue when cells and tumors are irradiated with charged particles. Thus, a better understanding of the dialogue between tumor cells and endothelial cells could help us to find new therapeutic targets that could be blocked to enhance radiotherapy effectiveness.

For these reasons, we decided to design new irradiation chambers allowing co-culture study in order to investigate the interplay between tumor cells and endothelial cells after alpha particle and proton irradiation. The aim of this work is to identify new radioresistance mechanisms that could be disrupted to increase radiation effects on tumor.

## 2. Results

In order to recover one type of cells from the irradiation chamber without cross-contamination from the other cell types and to perform the analyses described in this work, the solution we chose was to seed the two types of cells on different Mylar foils.

**Figure 1:**
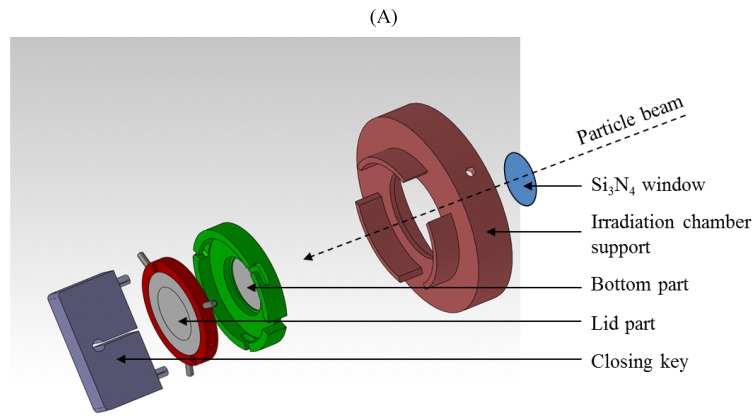

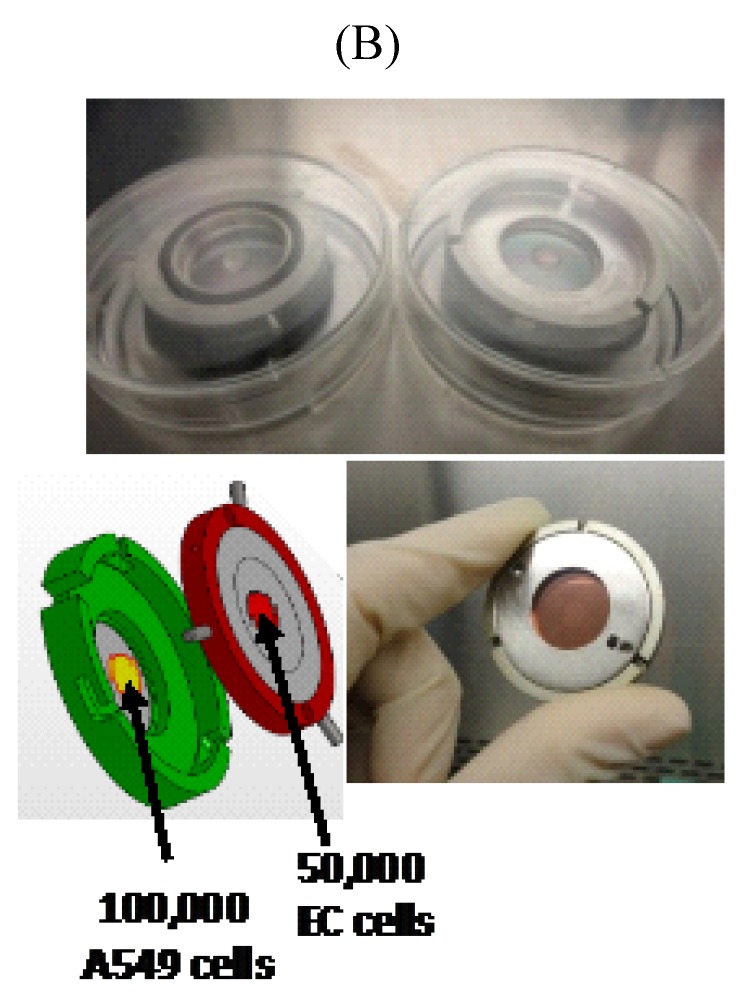
Schematic representation of co-culture irradiation chambers allowing indirect co-culture study (A). Detailed views of the bottom part and of the lid part (B).

We also chose this set up since *in vivo* in the tumors, endothelial cells are separated from the cancer cells by the basal membrane. Hence most of the dialog that exists between these two cell types is through diffusible molecules and not via cell-to-cell contacts. For each experiment, eight irradiation configurations were tested: A549 cells in monoculture irradiated or not (A549* or CTL A549), EC in monoculture irradiated or not (EC* or CTL EC), non-irradiated co-culture of both cell types (CTL A549/EC), co-culture irradiation of both cell types (A549*/EC*), co-culture where only A549 cells were irradiated (A549*/EC) and co-culture where only EC were irradiated (A549/EC*). 1–2 Gy alpha and 1.5 Gy proton irradiations were chosen. The RBE in comparison to X-rays has been determined in our experimental settings. The RBE for alpha particles (SF = 10%), with a LET of 100 keV/µm, is 5.5 [15] while the RBE 25 keV/µm proton beam is 3.2 [16]. Hence, 1.5 Gy of proton beam corresponds to 2.57 Gy of alpha particles, which is the same range as what we performed in this work. These doses were chosen because they induced changes in mRNA levels of genes of interest without too much effect on cell death as measured 24 h post-irradiation, at the time when mRNA levels were evaluated. Lower doses have no or very few effects on gene expression [15] while higher doses kill the cells. Most of the assays were performed 24 h after irradiation to permit communication between the two cell types. The co-culture configuration (Figure 1) allows indirect dialog between the two cell types via molecules secreted by one or the other cell type but does not permit gap junction-mediated communication.

### 2.1. Survival Fraction of A549 Cells and EC after Particle Irradiation in Mono- or Co-Culture Configurations

**Figure 2:**
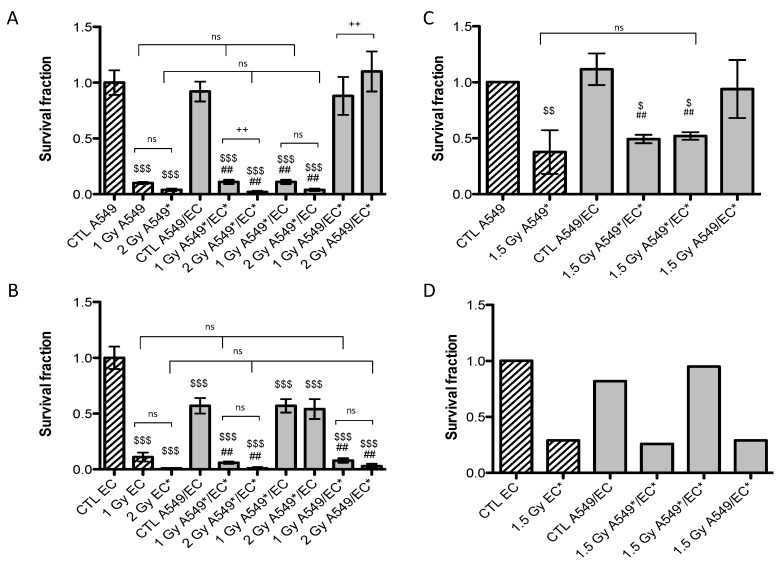
Survival fraction of A549 cells (**A**,**C**) and EC (**B**,**D**) exposed to alpha particles (**A**,**B**) or to proton beam (**C**,**D**) in mono- (hatched columns) or co-culture configurations (filled columns). Survival fraction was calculated using conventional clonogenic assays. Monoculture controls were set to one and all other configurations were normalized with monoculture controls. Results are presented as means ± 1 S.D. (A, B, C: three independent experiments with n = 3, D: two independent experiments with n = 2). * = irradiated cells. One-way ANOVA and Tukey’s multiple comparison post-test: ++ *p* < 0.01; +++ *p* < 0.001. Comparison with monoculture controls: $$ *p* < 0.01; $$$ *p* < 0.001; comparison with co-culture controls: ## *p* < 0.01; ### *p* < 0.001. ns: nonsignificant.

Twenty-four hours after exposure to a single dose of 1 Gy or 2 Gy of alpha particles or 1.5 Gy of proton beam, both cell types were plated for conventional clonogenic survival assays in order to compare their survival fraction in monoculture and in co-culture configurations (Figure 2). After exposure to a single dose of 1 Gy of alpha particles in monoculture configuration, the survival fraction of A549 cells fell to 10%. It dropped to 4% when irradiated with 2 Gy of alpha particles (Figure 2A) and to 37% when irradiated with 1.5 Gy of proton beam (Figure 2B). These results are in agreement with our previous results [15,17,18]. Similar survival fractions were obtained when A549 cells were irradiated in co-culture. There was no statistically significant difference between A549 cells irradiated in monoculture or in co-culture with EC. However, we observed a highly statistical significant difference between survival fractions of non-irradiated A549 cells co-cultivated with EC exposed to 2 Gy of alpha particles in comparison with the same configuration irradiated at 1 Gy or with the co-culture control (Figure 2A). This effect was not observed for proton irradiation.

The survival fraction of EC in monoculture exposed to 1 Gy of alpha particles was estimated to be 11% and decreased to 1% when irradiated with 2 Gy of alpha particles (Figure 2B). It was decreased to 29% when irradiated with 1.5 Gy of proton beam (Figure 2D). Once again, these results are in agreement with our previous ones [15]. There was no statistical significant difference between EC irradiated in monoculture compared with EC irradiated in co-culture configurations, whatever the dose or the particle used. However, when non-irradiated EC are co-cultivated with A549 cells, their survival fraction decreased to 57% but the co-cultivation with A549 cells did not change survival fraction of EC once irradiated.

### 2.2. Cell Cycle Analysis of A549 Cells and EC after Particle Irradiation in Mono- or Co-Culture Configurations

**Figure A1:**
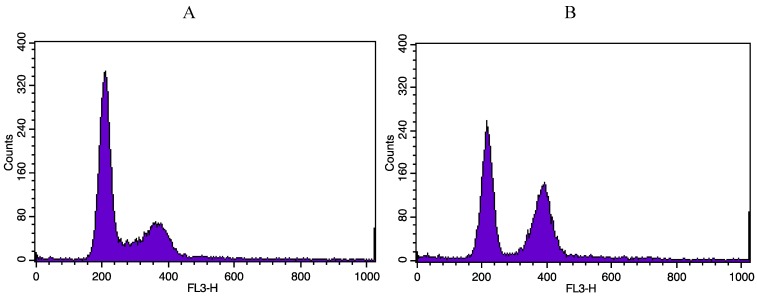
Illustrative cytometry graphs obtained for monocultured A549 cells stained with propidium iodide after irradiation with 1 Gy alpha particles. (**A**) proportion of non-irradiated A549 cells in the different cell cycle phases. (**B**) proportion of A549 cells in the different cell cycle phases 24h after irradiation with 1 Gy alpha particles.

**Figure A2:**
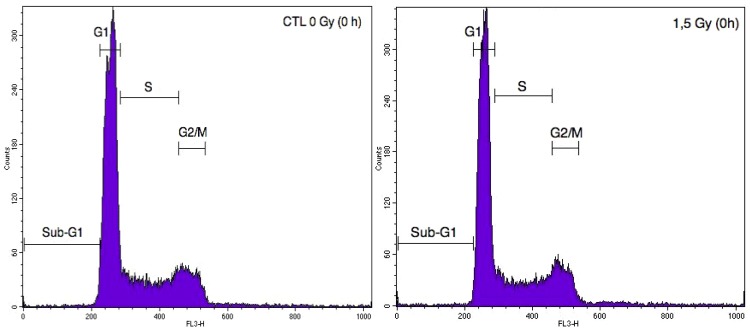

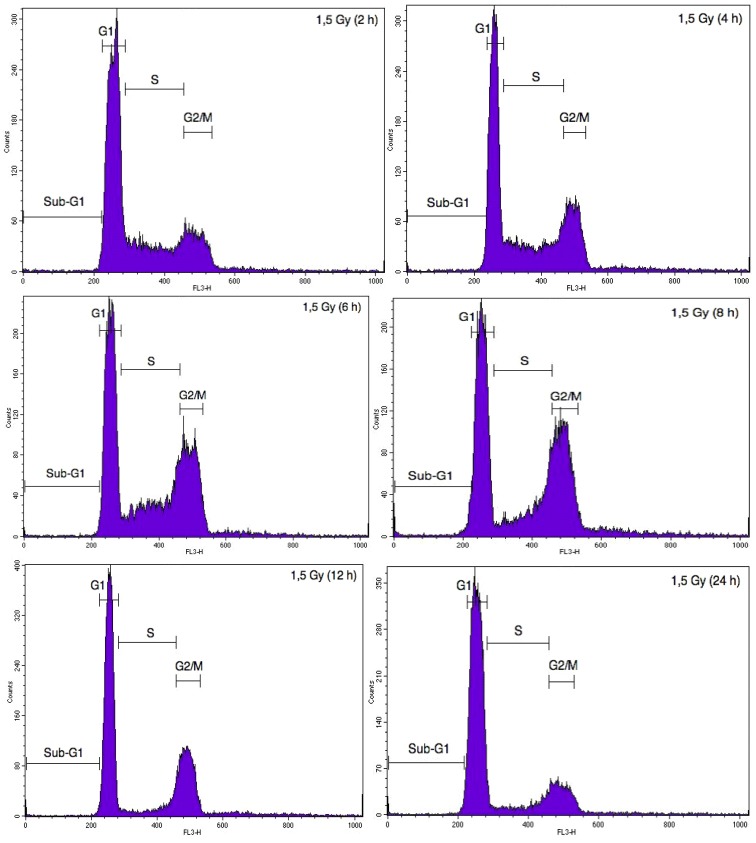
Illustrative cytometry graphs obtained for monocultured A549 cells stained with propidium iodide after irradiation with 1.5 Gy proton beam.

DNA damage induced by ionizing radiations triggers cell cycle arrest in G2/M phase to allow DNA repair in order to avoid entry of the cell into mitosis with damaged DNA [19]. In order to assess the proportion of A549 cells and EC in each phase of the cell cycle after alpha particle and proton irradiation, cell DNA content was evaluated using propidium iodide staining and analysed by flow cytometry. Twenty-four hours after alpha particle irradiation of A549 cells, the proportion of A549 cells in G2/M phase was markedly increased (Figure A1). However, the kinetics of cell cycle arrest in G2/M was not the same after proton irradiation. Indeed, a peak in cell cycle arrest was observed at 8 h after the irradiation (Figure A2). We thus chose to assess the effect of irradiation in co-cultures 24 h after irradiation with alpha particles for 8 h after irradiation with proton beam. Cell cycle arrest in G2/M was induced to the same extent both in mono- and co-culture configurations (Table 1). We obtained the same results when EC were exposed to 1 Gy of alpha particles or to 1.5 Gy proton beam, even if this G2/M accumulation was less pronounced (Table 1).

**Table 1:** Proportion of A549 cells and EC in the different cell cycle phases 24 h after exposure to 1 Gy of alpha particles or 8 h after exposure to 1.5 Gy of proton beam in mono- or co-culture configurations. Cells were fixed, stained with propidium iodide and percentages of cells in sub-G1, G1, S and G2/M phases were determined by flow cytometry. * = irradiated cells.

	**Alpha particles**	**G1 (%)**	**S (%)**	**G2/M (%)**
**Mono-culture**	CTL A549	38.9	48.9	12.1
	1 Gy A549*	38.4	22.5	39.2
**Co-culture**	CTL A549/EC	51.3	40.6	8.6
	1 Gy A549*/EC*	44.9	32.2	22.9
	1 Gy A549*/EC	45.4	30.2	24.1
	1 Gy A549/EC*	50.8	44.9	4.3
	**Proton beam**	**G1 (%)**	**S (%)**	**G2/M (%)**
**Mono-culture**	CTL A549	46.4	51.2	2.3
	1 Gy A549*	22.9	46.7	30.4
**Co-culture**	CTL A549/EC	50.3	45.2	4.5
	1 Gy A549*/EC*	27.9	45.8	26.3
	1 Gy A549*/EC	36	50.5	13.4
	1 Gy A549/EC*	47.6	51.1	1.3
	**Alpha particles**	**G1 (%)**	**S (%)**	**G2/M (%)**
**Mono-culture**	CTL EC	78	20.5	1.6
	1 Gy EC*	62.1	31.4	6.6
**Co-culture**	CTL A549/EC	66.4	30.8	2.8
	1 Gy A549*/EC*	69.8	19.2	11
	1 Gy A549*/EC	68.1	28.9	3
	1 Gy A549/EC*	66.5	28.9	4.6
	**Proton beam**	**G1 (%)**	**S (%)**	**G2/M (%)**
**Mono-culture**	CTL EC	72.9	23.3	3.7
	1 Gy EC*	68.1	24.9	7
**Co-culture**	CTL A549/EC	63	35.6	1.4
	1 Gy A549*/EC*	56.6	38	5.5
	1 Gy A549*/EC	62.4	34.8	2.9
	1 Gy A549/EC*	57	39.8	3.23

### 2.3. Effects of Particle Irradiation on Gene Expression in A549 Cells and EC in Mono- or Co-Culture Configurations

Radiation exposure of tumor cells is known to trigger several signal transduction pathways involved in cell survival, invasion and angiogenesis which modulate tumor response to radiation [20]. It has been demonstrated that irradiated tumor cells are able to protect endothelial cells from ionizing radiations by secreting growth factors and cytokines that trigger endothelial cell survival and migration [8,9,21]. This cross-talk between tumor cells and endothelial cells is implicated in radioresistance mechanisms and can highly influence the long-term tumor response to ionizing radiation [22]. In this context, we decided to investigate the effects of alpha particle irradiation on the expression of genes coding for growth factors, cytokines and proteins that could be implicated in the dialogue between A549 cells and EC and modulate tumor radioresistance. We used Taqman low-density array (TLDA) to perform the gene expression study on both cell types that were co-cultivated and exposed to 1 Gy of alpha particles. The complete dataset for A549 cells and EC are presented in Table A1. Nine genes were selected for their profile or because we had already observed their overexpression in a previous study [15]: *FAS* (CD95), *CCL2* (or MCP-1, monocyte chemotactic protein-1), *CCL5* (or RANTES), *CSF1* (colony stimulating factor-1), *IL8* (interleukin-8), *IL1B* (interleukin-1beta), *CXCL10* (or IP-10, interferon inducible protein-10), *FGF2* (or bFGF, fibroblast growth factor 2) and *PDGFB* (platelet-derived growth factor beta). We performed RT-qPCR assays for both cell types in mono- and co-culture configurations to confirm the changes in gene expression observed in our previous work and with TLDA.

**Figure 3:**
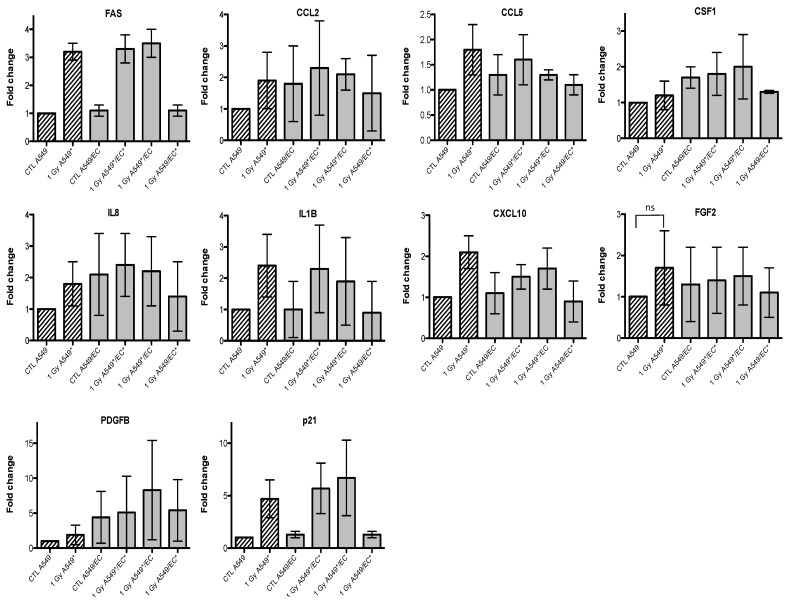
Effects of alpha particle (1 Gy) irradiation on the mRNA expression of *FAS*, *CCL2*, *CCL5*, *CSF1*, *IL8*, *IL1B*, *CXCL10*, *FGF2*, *PDGFB* and *p21* in A549 cells in mono- (hatched columns) or in co-culture (filled columns) configurations. Total RNA was extracted 24 h after irradiation and mRNA levels were quantified by RT-qPCR. Results are presented in induction fold as means ± 1 S.D. (n = 3) by comparison with non-irradiated cells in monoculture (CTL A549). * = irradiated cells. Repeated measures one-way ANOVA and Tukey post-test: + *p* < 0.05; +++ *p* < 0.001. Comparison with monoculture controls: $ *p* < 0.05; $$ *p* < 0.01; $$$ *p* < 0.001; comparison with co-culture controls: # *p* < 0.05; ### *p* < 0.001. ns: nonsignificant.

**Figure 4:**
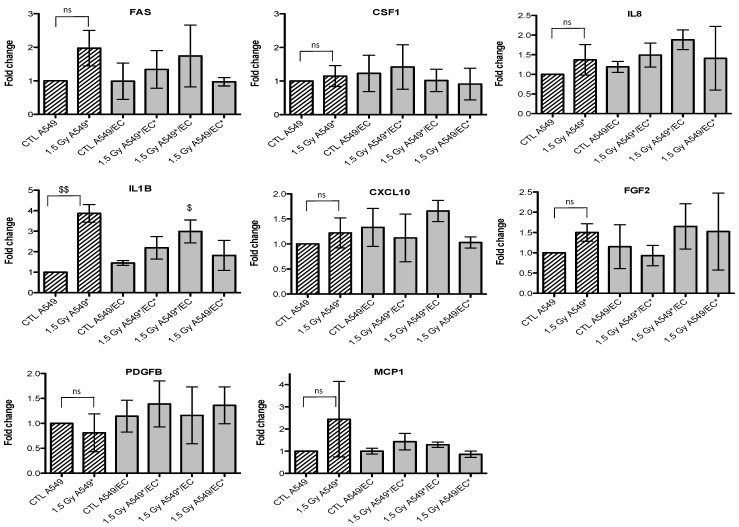
Effects of proton (1.5 Gy) irradiation on the mRNA expression of *FAS*, *CSF1*, *IL8*, *IL1B*, *CXCL10*, *FGF2*, *PDGFB* and *MCP1* in A549 cells in mono- (hatched columns) or in co-culture (filled columns) configurations. Total RNA was extracted 24h after irradiation and mRNA levels were quantified by RT-qPCR. Results are presented in induction fold as means ± 1 S.D. (n = 3) by comparison with non-irradiated cells in monoculture (CTL A549). * = irradiated cells. Repeated measures one-way ANOVA and Tukey post-test: comparison with monoculture controls: $ *p* < 0.05; $$ *p* < 0.01. ns: nonsignificant.

Given that we observed an accumulation of both cell types in G2/M phase, we also investigated the gene expression of p21 (or CDKN1, cyclin-dependent kinase inhibitor 1A), a well-known protein involved in cell cycle arrest. We observed an overexpression of *FAS* and *p21* after irradiation of A549 cells with alpha particles, no matter the configuration tested (Figure 3). A similar trend for *FAS* was observed after irradiation with proton beam (Figure 4).

**Figure 5:**
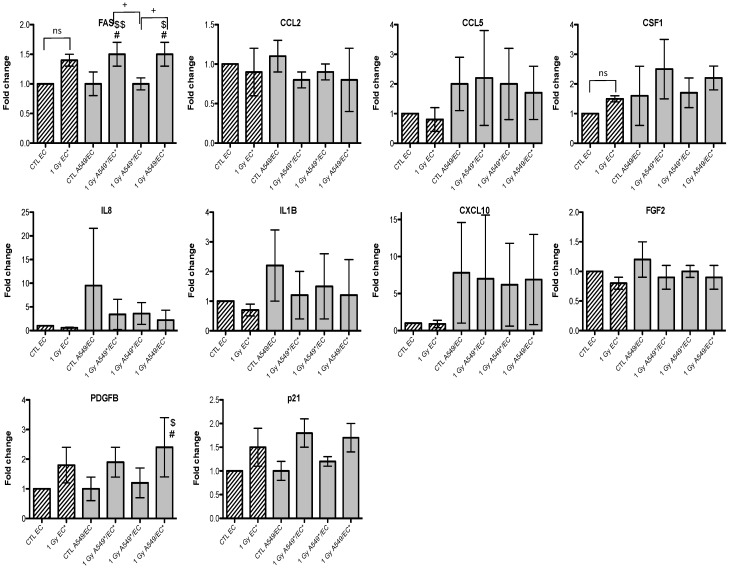
Effects of alpha particle (1 Gy) irradiation on the mRNA expression of *FAS*, *CCL2*, *CCL5*, *CSF1*, *IL8*, *IL1B*, *CXCL10*, *FGF2*, *PDGFB* and *p21* in endothelial cells in mono- (hatched columns) or in co-culture (filled columns) configurations. Total RNA was extracted 24h after irradiation and mRNA levels were quantified by RT-qPCR. Results are presented in induction fold as means ± 1 S.D. (n = 3) by comparison with non-irradiated cells in monoculture (CTL EC). * = irradiated cells. (Repeated measures one-way ANOVA and Tukey post-test: + *p* < 0.05; ++ *p* < 0.01. comparison with monoculture controls: $ *p* < 0.05; $$ *p* < 0.01; comparison with co-culture controls: # *p* < 0.05; ns: nonsignificant).

**Figure 6:**
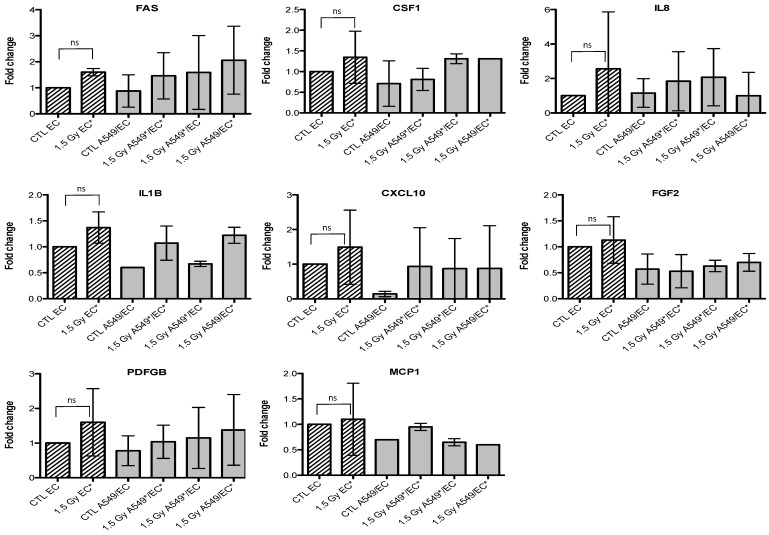
Effects of proton (1.5 Gy) irradiation on the mRNA expression of *FAS*, *CSF1*, *IL8*, *IL1B*, *CXCL10*, *FGF2*, *PDGFB* and *MCP1* in endothelial cells in mono- (hatched columns) or in co-culture (filled columns) configurations. Total RNA was extracted 24 h after irradiation and mRNA levels were quantified by RT-qPCR. Results are presented in induction fold as means (n = 2) by comparison with non-irradiated cells in monoculture (CTL EC). * = irradiated cells. Repeated measures one-way ANOVA and Tukey post-test: ns: nonsignificant.

Similar results were obtained with EC, even if the overexpression of *FAS* was smaller and *p21* overexpression did not reach statistical significance (Figure 5 and Figure 6). For the other genes of interest, little or no statistical significant changes were observed due to the high variability of the results observed from one experiment to another. However, *CXCL10*, for both types of irradiation, and *IL1B*, after alpha particle irradiation, had a similar expression profile as *FAS* in irradiated A549 cells. The gene expression of *CCL2*, *CSF1*, *IL8* and *PDGFB* seemed to be higher in co-cultivated A549 cells independently of irradiation compared to monoculture configurations. The same trend was observed in co-cultivated EC for *CCL5*, *IL8*, *IL1B* and *CXCL10* gene expression while gene expression profile for *PDGFB* and *CSF1* was similar to *FAS* gene expression profile.

## 3. Discussion

Given that a lot of cancer patients will receive radiotherapy during their treatment, any improvement in this field may increase the quality of life and the clinical outcome of many patients. The aim is to maximize the tumor cell killing while minimizing the side effects on normal tissues surrounding the tumor. Thanks to its advantages over X-rays, hadrontherapy is already used to treat tumors resistant to conventional radiotherapy, tumors localized near critical tissues and in paediatric oncology [23,24]. However, despite a growing interest in particle radiation therapies [25,26], the biochemical and biological effects of high-LET radiation is not yet fully understood. Indeed, due to a lack of randomised phase III clinical trials and the limited data available on this subject, little is known about the effects of heavy charged particle radiation on tumor response in terms of effectiveness, secondary cancers induction, metastasis development and angiogenesis [5,27]. Moreover, it is well known that tumor cells can actively protect their own vasculature from radiation damages that leads to enhance tumor radioresistance. The secretion of growth factors, such as vascular endothelial growth factor (VEGF) and FGF2, by irradiated tumor cells can protect endothelial cells from apoptosis induced by ionizing radiation [8,28]. In mice models bearing MCA/129 fibrosarcoma or B16F1 melanoma, it has been demonstrated that apoptosis resistance of the tumor vasculature led to an enhanced tumor growth and a decrease in radiotherapy efficacy [7]. Thus, understanding the mechanisms of radioresistance taking place during and after radiotherapy might open new ways to improve radiation efficacy [29]. Therefore, we decided to study the interplay between tumor cells and endothelial cells after alpha particle irradiation with the aim of finding new therapeutic molecules that could be targeted to enhance radiotherapy efficacy. We developed new irradiation chambers allowing indirect co-culture study in order to investigate the effect of alpha particle irradiation on a lung tumor cell line and endothelial cells in terms of cell survival, cell cycle arrest and gene expression changes.

First of all, clonogenic assays were performed on both cell types in mono- and co-culture configurations after alpha particle or proton irradiation. In monoculture, similar survival fractions were observed for both cell types exposed to the two types of particles compared to our previous study [15]. However, we did not observe any difference in cell survival of irradiated A549 cells and irradiated EC between mono- and co-culture configurations. This suggests that, in our model, A549 cells and EC could not protect each other from ionizing radiation. Nevertheless, EC exposed to 2 Gy alpha particles could enhance proliferation of non-irradiated A549 cells while co-cultivation of non-irradiated EC with A549 cells decreased the survival fraction of EC. These results suggest the existence of a dialogue between the two cell types but, in our study, no radioprotection was observed. As the number of cells in our set-up is limited, one possible explanation is that potential soluble radioprotective factors were too diluted in the medium and were not effective enough to trigger radioprotective mechanisms. Reducing the distance between the two Mylar foils, hence downsizing the volume of medium, would reduce the dilution of the molecules and possibly trigger much stronger effects. The design of new irradiation chambers with these characteristics is ongoing.

It is also possible that inter-cellular radioprotection mechanisms would not be sufficient to counteract the complex and harmful damages induced by alpha particles or proton beam. In this case, it is conceivable that high-LET heavy ions irradiations overcome radioresistance mechanisms. It has been demonstrated that high-LET heavy ions irradiation could overcome the radioresistance of anti-apoptotic Bcl-2 overexpressing tumor cells [30]. Finally, radioresistance mechanisms might need direct cell-to-cell contact to occur, such as gap junction intercellular communication [31].

Cell cycle arrest is a well-known process triggered by ionizing radiation in order to repair DNA damages before re-entry into mitosis. This is an important step in the DNA damage response because it prevents segregation of damaged chromosomes and propagation of errors to daughter cells [3]. Thus, we investigated the cell cycle distribution of each cell type after alpha particle and proton irradiation in mono- or co-culture configurations. We did not observe any difference between cells irradiated in mono- or co-culture but the proportion EC in the G2/M phase 24 h after irradiation was lower than the proportion of A549 cells in the same phase. This suggests that EC are able to repair their damages and resume cell division faster than A549 cells. This phenomenon has already been demonstrated by our group for alpha particles [32]. The repair kinetics of DNA double strand breaks by EC compared to A549 cells is cell cycle phase dependent and is about 10% faster for G1, 61% faster for S and 64% faster for G2 phase. It is not surprising since defects in DNA damages detection and in activation of the repair machinery is usually found in cancer cells leading to genomic instability, one of the enabling characteristic of cancer [6].

Finally, we investigated the gene expression changes induced by particle irradiation on both cell types in mono- and co-culture configurations. The aim was to highlight cytokines, growth factors or proteins that could be differentially overexpressed when cells were irradiated alone or in co-culture in order to point out new molecules that could be implicated in inter-cellular radioprotective mechanisms. Overexpression of *FAS* and *p21* genes was observed after irradiation of A549 cells or EC but it was independent of the mono- or co-culture configuration. *FAS* and *p21* genes are two well-known targets of the transcription factor p53, one of the central players implicated in the DNA damage response [33]. The *p21* gene overexpression confirmed the cell cycle analysis as this protein involved in cell cycle arrest was less expressed in EC compared with A549 cells whose proportion in G2/M phase was higher compared with EC. Changes in the expression of different cytokines and growth factors were also observed, that could be implicated in inter-cellular radioprotective communication but, because of a high variation between triplicates observed for these genes, it was difficult to draw any firm conclusions. However, it seems that *CXCL10* and *IL1B* gene overexpression in A549 cells had a similar expression profile than that of *FAS* suggesting that these genes may be involved in A549 cell response to ionizing radiation. Some pro-inflammatory and pro-angiogenic genes, such as *CCL2*, *CSF1*, *IL8* and *PDGFB*, seemed to be overexpressed by the co-culture configuration no matter if the cells were irradiated or not. It is however interesting to note that two studies reported angiogenic suppressive effects after irradiation of cancer cells with protons [34] or carbon ions [35]. In EC, *CCL5*, *IL8* and *CXCL10* genes were overexpressed independently of irradiation and *PDGFB* and *CSF1* gene overexpression had also similar profile to the one of *FAS*. Further investigations are needed to validate these results.

## 4. Materials and Methods

### 4.1. Cell Culture 

A549 cells and human endothelial EAhy926 cells were sub-cultured in 75-cm^2^ polystyrene flasks (Costar, Lowell, MA, USA) respectively in modified Eagle’s medium and Dulbecco’s Modified Eagle Medium (4.5 g/L D-glucose, Invitrogen, Carlsbad, CA, USA), all supplemented with 10% fetal calf serum (Invitrogen). EAhy926 cells are derived from fusion of human umbilical vein endothelial cells (HUVEC) with A549 cells. All cell types were cultured under an atmosphere containing 5% CO_2_.

### 4.2. Irradiation and Co-Culture Irradiation Chambers

A 2 MV Tandem accelerator (High Voltage Engineering Europa) of the University of Namur was used to obtain a He^2+^ and H^+^ homogenous broad beam over 1 cm^2^. The experimental set-up and the irradiation procedure are described elsewhere [16]. The linear energy transfer (LET) of the alpha particles was set up to 100 keV/µm because it corresponds to their maximum relative biological effectiveness (RBE) and the dose rate was 1 Gy/min. The linear energy transfer (LET) of the protons was set up to 25 keV/µm and the dose rate was 1 Gy/min [17]. The energy of the beam was tuned in order to deliver this LET within the cells to be irradiated.

For the co-culture study, the experimental set-up was modified. The beam exits in the air through a 1 µm thick Si_3_N_4_ foil, and the irradiation chambers are placed in front of the beam, at 3 mm from the exit window on an irradiation chamber support. The co-culture irradiation chambers were designed in such a way that two cell types can be seeded in the system to allow indirect co-culture study. The co-culture irradiation chamber consists of two 3 μm Mylar foils glued to two stainless steel pieces: the bottom part and the lid part separated by 5 mm of culture medium (2 mL total volume) (Figure 1). Twenty-four hours before irradiation, 100,000 A549 cells and 50,000 EC were seeded as a 32 µL drop on the Mylar foil on the bottom part and the lid part respectively. Cells were then allowed to adhere to the Mylar foil for 30 min and 2 mL of medium were added to each part. The cells were let alone for 24 h. Just before the irradiation, the chamber is closed so that, from now, cells were co-cultured with common shared media of 2 mL. The surface to be irradiated is 0.5 cm^2^. It has to be mentioned that only the cells on the side of the chamber that is closed to the Si_3_N_4_ foil are irradiated, the energy of the beam does not permit to irradiate the other side of the chamber. When the cells on the two sides of the chamber have to be irradiated, the chamber is turned in between the two irradiations so that the cells from the far side come closed to the Si_3_N_4_ foil and the cells that have just been irradiated come on the far side. Most of the experiments were performed twenty-four hour after the irradiation to permit communication between the two cell types. For that the irradiation chambers are put back in the CO_2_ incubator.

### 4.3. Clonogenic Assay

Cells were trypsinized, counted and replated for clonogenic survival assay at appropriate cell numbers in 6-well plates. The entire procedure after irradiations has been previously described [16]. As EC could not grow properly when plated at low densities, we used fresh medium plus 10% serum supplemented with conditioned medium 50% as recommended by Franken *et al.* [36]. Three independent experiments were performed for each irradiated cell type and the errors were evaluated as standard deviation. One-way ANOVA and Tukey’s multiple comparison post-test were performed with GraphPad Prism version 5.02 for Windows (GraphPad Software, San Diego, CA, USA, www.graphpad.com). Data are presented as means ± 1 S.D.

### 4.4. Cell Cycle Analysis

In brief, trypsinized cells were fixed with ice-cold 70% ethanol at −20 °C during at least one hour. After multiple centrifugations and blocking steps with PBS plus 10% fetal calf serum, cells were treated with RNAse A (50 µg/mL) and 0.1% tween in PBS during 30 min at 37 °C. Propidium iodide staining (20 µg/mL) was performed at 4 °C during 10 min in the dark. The percentages of G1, S, and G2/M phase cells were determined by the Modfit software, following flow cytometry analysis (FACSCalibur, BD Biosciences, Franklin Lakes, NJ, USA) where 8,000 or more cells were analyzed for each run.

### 4.5. Gene Expression Analysis on TaqMan Low-Density Array

Total RNA was extracted using the Total RNAgent extraction kit (Promega, Madison, WI, USA). The “High Capacity cDNA Archive” kit from Applied Biosystems (Carlsbad, CA, USA) was used to reverse transcribed 1 μg total RNA or 250 ng of total RNA for A549 cells and EC respectively. PCR amplifications on TLDA Human Immune were performed in a 7900HT Fast Real-Time PCR system. Relative fold-inductions were calculated by the comparative cycle threshold method (2^−ΔΔCt^). To determine the most appropriate endogenous control, we used Genorm on the six housekeeping genes. The housekeeping gene used for normalization was GAPDH.

### 4.6. Quantitative Real-Time PCR

Total RNA was extracted with the RNeasy kit (Qiagen, Hilden, Germany). At least 500 ng of RNA was reverse transcribed into cDNA using SuperScript II Reverse Transcriptase (Invitrogen). Amplification reaction assays contained SYBR Green PCR Mastermix (Applied Biosystems) and primers at the optimal concentrations. The relative fold-induction was calculated by the cycle threshold method and the housekeeping gene used for normalization was GAPDH. Experiments were performed in triplicates. Repeated measures one-way ANOVA and Tukey post-test were performed with GraphPad Prism version 5.02 for Windows. Data are presented as means ± 1 S.D.

## 5. Conclusions

In summary, we have developed a new irradiation chamber allowing charged particle irradiation of co-cultured cell lines. We decided to study the interplay between tumor cells and endothelial cells after alpha particle irradiation but we could not observe any radioprotection of one cell type on the other. However, we did observe that irradiation with alpha particles induced expression changes of genes implicated in inflammation, cell death and angiogenesis that could modify tumor radiosensitivity. This notably the case for *FAS*, *p21*, *CXCL10* and *IL1B*. The comprehension of radioresistance mechanisms taking place during and after ionizing treatment is essential to discover new interplay pathways that could be targeted during radiotherapy in order to enhance its effectiveness.

## Appendix

**Table A1:** Gene expression profiles of co-cultivated A549 cells and EC exposed to alpha particles (1 Gy). Total RNA was extracted 24 h after radiation exposure and retrotranscribed before being hybridized onto TaqMan low-density array human immune as described in Materials and Methods. Results are presented in induction fold by comparison with the reference condition. both cell types non-irradiated. NE: not expressed *i.e.*, Ct value > 35 or undetermined. * = irradiated cells.

Gene Symbol	A549 cell gene expression			EC gene expression		
	1 Gy A549*/EC*	1 Gy A549*/EC	1 Gy A549/EC*	1 Gy A549*/EC*	1 Gy A549*/EC	1 Gy A549/EC*
*ACE*	NE	1.2	0.8	NE	NE	NE
*AGTR1*	NE	NE	NE	NE	NE	NE
*AGTR2*	NE	NE	NE	NE	NE	NE
*BAX*	0.8	2.1	0.9	1.1	1.0	1.6
*BCL2*	1.0	1.1	2.0	NE	1.1	NE
*BCL2L1*	2.5	2.7	0.6	1.5	1.2	2.1
*C3*	2.1	2.2	0.7	NE	NE	NE
*CCL19*	NE	NE	NE	NE	NE	NE
*CCL2*	2.0	2.4	5.2	1.2	1.5	1.3
*CCL3*	NE	NE	NE	NE	NE	NE
*CCL5*	1.8	2.0	1.2	1.5	3.9	0.9
*CCR2*	NE	NE	NE	NE	NE	NE
*CCR4*	NE	NE	NE	0.5	0.7	0.8
*CCR5*	NE	NE	NE	NE	NE	NE
*CCR7*	NE	NE	NE	NE	NE	NE
*CD19*	NE	NE	NE	NE	NE	NE
*CD28*	NE	NE	NE	NE	NE	NE
*CD34*	NE	NE	NE	0.8	NE	1.3
*CD38*	1.9	2.1	0.9	NE	NE	NE
*CD3E*	NE	NE	NE	NE	NE	NE
*CD4*	NE	NE	NE	NE	NE	NE
*CD40*	1.4	2.4	1.8	1.2	0.9	1.4
*CD40LG*	NE	NE	NE	NE	NE	NE
*CD68*	1.2	2.0	1.0	0.9	1.0	1.4
*CD80*	NE	NE	NE	NE	NE	NE
*CD86*	NE	NE	NE	NE	NE	NE
*CD8A*	0.5	0.5	0.5	NE	NE	NE
*COL4A5*	2.0	2.3	0.6	1.0	0.9	1.3
*CSF1*	2.0	2.1	1.0	1.3	1.6	1.3
*CSF2*	9.5	3.4	2.7	0.9	0.9	1.1
*CSF3*	NE	NE	NE	1.5	1.7	1.8
*CTLA4*	NE	NE	NE	NE	NE	NE
*CXCL10*	3.4	4.4	1.1	10.5	5.5	7.8
*CXCL11*	1.2	2.1	0.9	7.3	4.7	3.2
*CXCR3*	NE	NE	NE	NE	NE	NE
*CYP1A2*	NE	NE	NE	NE	NE	NE
*CYP7A1*	NE	NE	NE	NE	NE	NE
*ECE1*	1.2	1.3	0.6	0.9	0.8	1.1
*EDN1*	1.4	1.3	0.9	1.0	0.8	1.0
*FAS*	6.4	5.0	1.0	1.4	0.8	1.2
*FASLG*	NE	NE	NE	NE	NE	NE
*FN1*	1.1	1.4	0.9	1.4	2.1	1.3
*GNLY*	NE	NE	NE	NE	NE	NE
*GZMB*	NE	NE	NE	NE	NE	NE
*HLA-DRA*	NE	1.1	1.0	0.9	0.8	1.0
*HLA-DRB1*	NE	NE	NE	NE	NE	NE
*HMOX1*	0.0	1.3	1.1	1.8	2.1	1.2
*ICAM1*	NE	1.7	1.1	0.8	0.8	0.5
*ICOS*	NE	NE	NE	NE	NE	NE
*IFNG*	NE	NE	NE	NE	NE	NE
*IKBKB*	0.8	0.8	0.6	0.7	0.9	0.8
*IL10*	NE	NE	NE	NE	NE	NE
*IL12A*	1.7	0.9	0.9	1.9	1.3	1.4
*IL12B*	NE	NE	NE	NE	NE	NE
*IL13*	NE	NE	NE	NE	NE	NE
*IL15*	NE	NE	NE	1.1	1.1	NE
*IL17*	NE	NE	NE	NE	NE	NE
*IL18*	1.3	2.1	2.1	0.7	0.8	1.3
*IL1A*	2.6	2.5	1.5	0.9	0.8	0.7
*IL1B*	3.0	3.1	0.7	0.8	1.0	0.6
*IL2*	NE	NE	NE	NE	NE	NE
*IL2RA*	NE	NE	NE	NE	NE	NE
*IL3*	NE	NE	NE	NE	NE	NE
*IL4*	NE	NE	NE	NE	NE	NE
*IL5*	NE	NE	NE	NE	NE	NE
*IL6*	3.1	3.1	1.3	1.1	1.1	1.2
*IL7*	NE	NE	NE	1.1	1.3	NE
*IL8*	1.8	1.7	1.0	0.5	1.5	0.4
*IL9*	NE	NE	NE	NE	NE	NE
*LRP2*	NE	NE	NE	NE	NE	NE
*LTA*	NE	NE	NE	8.2	5.8	8.7
*MYH6*	NE	NE	NE	NE	NE	NE
*NFKB2*	1.4	1.4	0.9	0.8	1.1	0.6
*NOS2A*	NE	NE	NE	NE	NE	NE
*PRF1*	NE	NE	NE	NE	NE	NE
*PTGS2*	NE	NE	NE	NE	NE	NE
*PTPRC*	NE	NE	NE	NE	NE	NE
*REN*	NE	NE	NE	NE	NE	NE
*RPL3L*	NE	NE	NE	0.4	0.3	0.4
*SELE*	NE	NE	NE	NE	NE	NE
*SELP*	NE	NE	NE	0.9	0.8	0.9
*SKI*	0.8	1.0	0.9	0.7	0.9	1.2
*SMAD3*	0.7	0.6	0.4	1.0	0.9	1.4
*SMAD7*	0.9	1.1	1.2	0.5	0.9	0.4
*STAT3*	1.3	1.6	0.8	1.0	0.9	1.5
*TBX21*	NE	NE	NE	NE	NE	NE
*TGFB1*	1.0	1.2	0.8	NE	NE	NE
*TNF*	1.6	1.1	NE	NE	NE	NE
*TNFRSF18*	NE	NE	NE	NE	NE	NE
*VEGF*	1.2	1.4	1.0	1.2	1.0	1.0
